# Molecular identification and physiological functional analysis of NtNRT1.1B that mediated nitrate long-distance transport and improved plant growth when overexpressed in tobacco

**DOI:** 10.3389/fpls.2023.1078978

**Published:** 2023-02-28

**Authors:** Changzheng Wu, Yucheng Xiang, Pingjun Huang, Mingfa Zhang, Ming Fang, Weiqin Yang, Wenrui Li, Fengchun Cao, Lai-Hua Liu, Wenxuan Pu, Shuhui Duan

**Affiliations:** ^1^ College of Resources and Environmental Sciences, Department of Plant Nutrition, Key Lab of Plant-Soil Interaction of Ministry of Education, China Agricultural University, Beijing, China; ^2^ Tobacco Research Institute of Technology Centre, China Tobacco Hunan Industrial Corporation, Changsha, China; ^3^ Hunan Tobacco Research Institute (Changsha, Chenzhou, Xiangxi), China National Tobacco Corporation Hunan Company, Changsha, China

**Keywords:** nitrate transporter NtNRT1.1B, yeast complementation, long-distance transport, promoter activity, overexpression in tobacco, nitrogen use efficiency

## Abstract

Although recent physiological studies demonstrate that flue-cured tobacco preferentially utilizes nitrate ( ${\text{NO}}_{3}^{-}$ ) or ammonium nitrate (NH_4_NO_3_), and possesses both high- and low-affinity uptake systems for ${\text{NO}}_{3}^{-}$ , little is known about the molecular component(s) responsible for acquisition and translocation in this crop. Here we provide experimental data showing that *NtNRT1.1B* with a 1,785-bp coding sequence exhibited a function in mediating ${\text{NO}}_{3}^{-}$ transport associated with tobacco growth on ${\text{NO}}_{3}^{-}$ nutrition. Heterologous expression of *NtNRT1.1B* in the ${\text{NO}}_{3}^{-}$ uptake-defective yeast *Hp△ynt1* enabled a growth recovery of the mutant on 0.5 mM ${\text{NO}}_{3}^{-}$ , suggesting a possible molecular function of *NtNRT1.1B* in the import of ${\text{NO}}_{3}^{-}$ into cells. Transient expression of NtNRT1.1B::green fluorescent protein (GFP) in tobacco leaf cells revealed that NtNRT1.1B targeted mainly the plasma membrane, indicating the possibility of ${\text{NO}}_{3}^{-}$ permeation across cell membranes *via* NtNRT1.1B. Furthermore, promoter activity assays using a GFP marker clearly indicated that *NtNRT1.1B* transcription in roots may be down-regulated by N starvation and induced by N resupply, including ${\text{NO}}_{3}^{-}$ , after 3 days’ N depletion. Significantly, constitutive overexpression of *NtNRT1.1B* could remarkably enhance tobacco growth by showing a higher accumulation of biomass and total N, ${\text{NO}}_{3}^{-}$ , and even ${\text{NH}}_{4}^{+}$ in plants supplied with ${\text{NO}}_{3}^{-}$ ; this NtNRT1.1B-facilitated N acquisition/accumulation could be strengthened by short-term ^15^N- ${\text{NO}}_{3}^{-}$ root influx assays, which showed 15%–20% higher ${\text{NO}}_{3}^{-}$ deposition in *NtNRT1.1B*-overexpressors as well as a high affinity of NtNRT1.1B for ${\text{NO}}_{3}^{-}$ at a *K*_m_ of around 30–45 µM. Together with the detection of *NtNRT1.1B* promoter activity in the root stele and shoot–stem vascular tissues, and higher ${\text{NO}}_{3}^{-}$ in both xylem exudate and the apoplastic washing fluid of *NtNRT1.1B*-transgenic lines, *NtNRT1.1B* could be considered as a valuable molecular breeding target aiming at improving crop N-use efficiency by manipulating the absorption and long-distance distribution/transport of nitrate, thus adding a new functional homolog as a nitrate permease to the plant NRT1 family.

## Introduction

Nitrate ( ${\text{NO}}_{3}^{-}$ ) represents a major inorganic nitrogen (N) species absorbed preferentially by most plants in dryland soils (Marschner, 1995). To achieve higher yields in modern agriculture, a large annual amount of N fertilizers are increasingly used to generate higher crop production, but, on average, most crops recover less than 40% of supplied N (Good and Beatty, 2011). This demonstrates a low N-use efficiency (NUE) in farming practice, which is undoubtedly associated with great economic loss and environmental damage. Thus, intensively exploring and understanding genetic bases that assist the molecular breeding of new varieties, with an improved ability to effectively acquire and use soil N, has been an attractive long-term goal in agriculture (Good et al., 2004; Hirel et al., 2007).

To date, mechanisms of nitrate uptake from external media, including from soils *via* roots, nitrate’s translocation within the plant, and N metabolic processes have been comprehensively investigated (Nacry et al., 2013; O'Brien et al., 2016). So far, the transporters/channels in plants for ${\text{NO}}_{3}^{-}$ have been assigned to members of four different families, namely NPF, the nitrate transporter 1/peptide transport family (NRT1/PTR), the nitrate transporter 2 (NRT2) family, the chloride channel family (CLC), and the slow anion-associated channel homolog family (SLC/SLAH) (Léran et al., 2014; O'Brien et al., 2016). However, only certain members of the NRT1/NPF and NRT2 families have been confirmed to be responsible for ${\text{NO}}_{3}^{-}$ uptake from soils/external media (Nacry et al., 2013; Kant, 2018). These ${\text{NO}}_{3}^{-}$ transporters are topologically predicted to span a biological membrane 12 times (Léran et al., 2014; Liu et al., 2018) to facilitate the proton-coupled active movement of ${\text{NO}}_{3}^{-}$ (Chen et al., 2008), and most of the nitrate transporters (NRTs) characterized so far are either a high-affinity transport system (HATS; working normally at a concentration of less than 0.5 mM) or a low-affinity transport system (LATS; where ${\text{NO}}_{3}^{-}$ availability is > 0.5 mM) (Wang et al., 2012; O'Brien et al., 2016; Kant, 2018; Carillo and Rouphael, 2022).

Physiologically, multiple nitrate permeases of NRT1s and NRT2s respond to ${\text{NO}}_{3}^{-}$ at a transcriptional level and may act together to enable the effective absorption of N by plants, depending on tissues/organs, developmental phases, and environmental conditions (Wang et al., 2012; Liu et al., 2018). Some NRTs (e.g., AtNRT1.1/1.4/1.6/2.1) are either differentially or coordinately regulated by nitrate, N starvation/metabolites, sucrose, circadian rhythm, and pH (Krouk et al., 2010; Medici and Krouk, 2014; Liu et al., 2018). Molecularly, most NRT1-type transporters act as low-affinity ${\text{NO}}_{3}^{-}$ permeases, except for AtNRT1.1/MtNRT1.3, which are dual-affinity ${\text{NO}}_{3}^{-}$ transporters depending on the phosphorylation state of the T101 residue, which occurs at a very low concentration of ${\text{NO}}_{3}^{-}$ (Liu and Tsay, 2003; Morere-Le Paven et al., 2011). Intriguingly, AtNRT1.1 is demonstrated to be an ${\text{NO}}_{3}^{-}$ sensor, monitoring alterations in external ${\text{NO}}_{3}^{-}$ concentrations to promote an appropriate metabolic adaption as well as root-architectural reshaping, which is ascribed to a transport activity for auxin of AtNRT1.1 that is post-translationally regulated by severely low external ${\text{NO}}_{3}^{-}$ , thus named “transceptor” (Ho et al., 2009). In contrast to NRT1s, most identified NRT2 proteins show a stronger specificity for ${\text{NO}}_{3}^{-}$ with a high affinity, but such NRT2s alone do not exhibit ${\text{NO}}_{3}^{-}$ transport activity if lacking an interaction with the NAR2 protein (Wang et al., 2012). In *Arabidopsis*, NRT1.2 (NPF4.6/AIT1), a second nitrate permease characterized in plants, functions as a constitutive LATS for ${\text{NO}}_{3}^{-}$ movement across the cell membrane, with a *K*_m_ value of about 5.9 mM, and its expression occurs in the root epidermis and cortex (Huang et al., 1999). Interestingly, a further transport activity assay demonstrates that NRT1.2 serves as an abscisic acid (ABA) importer with a *K*_m_ ≈ 5 μM to regulate the stomatal aperture of upper parts (Kanno et al., 2012; Kanno et al., 2014), and that its stability and ABA transport activity are controlled by a C-terminally encoded peptide receptor 2 (CEPR2)-mediated phosphorylation event (Zhang et al., 2021). Although this activity seems to be nitrate independent, nitrate does alleviate ABA-mediated inhibition of seed germination in both the *NRT1.2* mutant and its wild type (WT) (Kanno et al., 2014), suggesting that *NRT1.2* would not be implicated in a direct physiological linkage between ${\text{NO}}_{3}^{-}$ and ABA signals.

As well as serving as a model plant for studying fundamental biological processes, common tobacco (*Nicotiana tabacum*), an allotetraploid plant species, represents one of the most cultivated non-food crops worldwide, having spread to more than 120 countries (Sierro et al., 2014). Its agricultural production requires a huge input of manufactured N fertilizers, with an annual world consumption of around 150,000 tons of net N estimated (Liu et al., 2018), but more than half of the applied N is lost into the environment, which is at least due in part to inefficient N uptake and utilization by this crop (Sisson et al., 1991). In addition, N nutrition greatly affects the composition and content of N-containing compounds (e.g., proteins, nicotine, and aromatic heterocyclic substances) in tobacco products; and such compounds’ biological activities involved in their molecular generation and decomposition are of great interest to biochemists (Hoffmann and Hoffmann, 1977). Recent physiological studies of growth phenotyping on different N sources and short-term root uptake assays using ^15^N- ${\text{NO}}_{3}^{-}$ tracer have clearly demonstrated that flue-cured tobacco (e.g. K326 and HD) preferentially utilizes ${\text{NO}}_{3}^{-}$ or ammonium nitrate (NH_4_NO_3_), but not ${\text{NH}}_{4}^{+}$ alone, with both high- and low-affinity uptake/transport processes for ${\text{NO}}_{3}^{-}$ at a concentration range of 2–1,000 µM and 1,000–7,000 µM, respectively (Fan et al., 2018). However, apart from early reports showing sequence isolation, transcriptional regulation, and functionality of *NRT2.1* from *Nicotiana plumbaginifolia* (Quesada et al., 1997; Krapp et al., 1998; Fraisier et al., 2000; Liu et al., 2018), little is described at a molecular level about ${\text{NO}}_{3}^{-}$ acquisition by and translocation/allocation within the common tobacco plant. Here we report the functional characterization of a nitrate transporter homolog, *NRT1.1B*, from *N. tabacum* L. cv. K326 (an internationally cultivated tobacco variety). By means of comprehensive approaches, including molecular cloning and a heterologous growth complementation test, marker protein-based subcellular localization and promoter activity analysis, isotopic ^15^N root influx measurement, transgenic effect assessment, and a long-distance transport assay of ${\text{NO}}_{3}^{-}$ , we show the physiological significance of *NtNRT1.1B* in ${\text{NO}}_{3}^{-}$ translocation and utilization for tobacco plant growth.

## Materials and methods

### Plant growth condition

Seeds of tobacco (*N. tabacum* L., cv. K326) were sterilized with 70% ethanol for 2 min and then with 2% sodium hypochlorite solution for a further 15–20 min, rinsed five times with sterile water, and germinated on 1/2 Murashige & Skoog (MS) agar plates for 14 days. Seedlings were transferred to a hydroponic or soil growth system in a growth room (16 h light/8 h dark cycle, 250 µE·m^–2^·s^–1^ light, 26/22°C light/dark temperature regime, 60% relative humidity). Plastic pots (15 cm × 15 cm × 20 cm) filled with 2.8 L of normal nutrient solution and others filled with 2 kg of soil (80% peat and 20% vermiculite, soil moisture 60%–70%) were used for the plant culture. The normal nutrient solution (for normal growth) contained 1 mM NH_4_NO_3_, 0.8 mM K_2_SO_4_, 1 mM KH_2_PO_4_, 1.5 mM MgSO_4_, 2 mM CaCl_2_, 3 µM MnSO_4_, 1 µM ZnSO_4_, 1 µM CuSO_4_, 0.1 µM (NH_4_)_6_Mo_7_O_4_·4H_2_O, 1 µM H_3_BO_4_, and 20 µM Fe–EDTA. The pH at 6.0–6.3 was adjusted using 1 M KOH.

For the test of growth on ${\text{NO}}_{3}^{-}$ as an N source, after the pre-culture of seedlings with the above solution at half-strength or soil for 14 days, plants of a similar size were grown for 25 days in the nutrient solution containing different ${\text{NO}}_{3}^{-}$ concentrations (0.5 mM, 2.0 mM, or 5.0 mM; aerated *via* an electric pump and refreshed every 2 days). The plants grown in the pot soil for 25 days were supplied three times with 100 mL of water containing ${\text{NO}}_{3}^{-}$ at a concentration of 0 mM, 0.5 mM, 2 mM, or 5 mM. Four biological replicates were conducted for each treatment.

### Phylogenetic analysis

Homologous sequences of *Arabidopsis* AtNRT1.1s or AtNPF6s were extracted from the ARAMEMNON database (http://aramemnon.uni-koeln.de/index.ep), tobacco NtNPF6s from a publication by Zhan et al. (2022), and NtNRT1.1 and NtNRT1.2 from Liu et al. (2018). The ClustalW method in the Molecular Evolutionary Genetics Analysis (MEGA) 7.0 software was used to perform a multiple sequence alignment of putative NPF6 peptides for constructing a phylogenetic tree and sequence percent identity, with the following parameters: a gap opening penalty of 15, a gap extension penalty of 0.3, a 25% delay of divergent sequences, and a Gonnet series as the protein weight matrix. The phylogenetic tree and percent identity of NPF6s was constructed with MEGA 7.0 software using the neighbor-joining algorithm. Bootstrap analysis was carried out with 1,000 replicates. Branch lengths (drawn in the horizontal dimension) are proportional to the phylogenetic distances.

### Cloning and yeast functional complementation

The putative open reading frame (ORF) of *NtNRT1.1B* (i.e., *NtNRT1.2* in Liu et al., 2018) was amplified by PCR using the specific primers containing the *SalI* site (in lowercase letters): NtNRT1.1B-SalI-F, 5′-ATTAgtcgacATGGCACTTCCTGAGACACA-3′, and NtNRT1.1B-SalI-R, 5′-ACTAgtcgacATGACAAACCGGTCCATC-3’. The ORF with the *SalI* overhang was ligated into the yeast expression vector pYNR (Montanini et al., 2006) after its linearization by *SalI*. The pYNR-*NtNRT1.1B* plasmid was transformed into the (*Hansenula polymorpha*) *YNT1* deletion yeast strain △ynt (*△ynt, △leu*), which is unable to grow on less than 0.5 mM ${\text{NO}}_{3}^{-}$ as a sole N source; the pYNR was, respectively, introduced into the yeast strain NCYC495 (*△leu*) (Pérez et al., 1997) and △ynt (*△ynt, △leu*) as a positive and negative control. Yeast transformation and complementation were performed, as described in Liu et al. (Liu et al., 2003 note: growth at 37°C). All transformants were first selected on NAAG agar medium (2% glucose, 2% agar form Oxid, 0.17% yeast nitrogen base without amino acids, and ammonium sulfate from Difco, Detroit, MI, USA) containing 5 mM NaNO_3_ as the N source. A single colony was picked, suspended in 60 μL of water, serially diluted, and spotted (2 μL) onto the NAAG agar media supplemented with 0.5 mM ${\text{NO}}_{3}^{-}$ as the sole N source. The medium pH was adjusted by 1 M HCl or KOH.

### NtNRT1.1B protein subcellular localization

The *NtNRT1.1B* ORF without a stop codon was amplified by a high-fidelity DNA polymerase (NEB, Beijing, China) using primers containing the *BamHI* site (NtNRT1.1B-BamHI-F, TTggatccATGGCACTTCCTGAGACA and NtNRT1.1B-BamHI-R, gtggatccG ACAATGACAAACC GGTCCAT), cloned into the vector pCF203 [carrying a cauliflower mosaic virus (CaMV) 35S promoter and the GFP gene (Liu et al., 2003)]. For the preparation of the transient expression of *NtNRT1.1B::GFP* in leaves of tobacco (*N. tabacum*), an overnight culture (OD_600_ = 0.6) of *Agrobacterium* GV3101 harboring *pCF203-35S*-*NtNRT1.1B*::*GFP* was centrifuged at 2,000 g for 10 min; the collected bacteria were resuspended in an infiltration medium consisting of 50 mM MES (4-morpholineethanesulfonic acid), 2 mM Na_3_PO_4_·12H_2_O, and 100 μM acetosyringone. Leaves of 19-day tobacco were infiltrated using a 2 mL syringe (without a needle) and grown in a greenhouse for 2–3 days; leaf cells were visualized by confocal microscopy after a 48- to 72-h infiltration.

For staining of the plasma membrane (PM), transfected tobacco leaves were incubated with 20 μM FM™ 4-64 [a molecular probe, N-(3-triethylammoniumpropyl)-4-(6-(4-(diethylamino) phenyl) hexatrienyl) pyridinium dibromide (Bolte et al., 2004) for 10 min and washed three times with sterile water before visualization. Plant cells were scanned with an energy excitation and emission wavelength at 488 nm and 543 nm by a confocal laser scanning microscope (LSM880, Zeiss, Germany). The PM signal (dyed by FM 4-64) was visualized at between 515 and 640 nm. Brightness and contrast pictures were adjusted using ZEN 2.3 SP1 software. Being a PM-targeted protein marker, the expression of *AtDUR3* (Liu et al., 2003) in leaf cells was tested as a reference.

### Histochemical analysis of *NtNRT1.1B* promoter activity

A putative promoter (Pro*_NtNRT1.1B_*) with a 2,000-bp genomic sequence upstream from the predicted translational start of *NtNRT1.1B* was amplified by PCR using primers consisting of the *HindIII* and *SalI* cloning site (5′-CCCaagcttTGGGAATTTG AAAATTCTACA-3′ and 5′-ACGcgtcgacTTTAGCAAC TTTTCTTGCAC-3′), and cloned into a binary expression vector pBI101-β-glucuronidase (*GUS*) and -*GFP* (harboring the kanamycin resistance gene for plant selection), yielding recombination plasmids containing the constructs “Pro*_NtNRT1.1B_*-*GUS*” and “Pro*_NtNRT1.1B_*-*GFP*”. These constructs were transformed into tobacco plants using the agrobacterial GV3101-mediated leaf-disk method, as described in Sparkes et al. (2006). Several transgenic lines of Pro*_NtNRT1.1B_*-*GUS*’ and ‘Pro*_NtNRT1.1B_*-*GFP* were generated based on kanamycin selection. GUS staining and microscopic (BX51, Olympus, Japan) visualization were performed as stated by Zhang et al. (2019). For the analysis of promoter activity in tissue sections, stained stems were further embedded in paraffin, cut into 10-µm-thick transverse sections, mounted on a glass slide, and visualized under the microscope.

For the observation of GFP localization, roots were mounted in water under a glass coverslip, and GFP signals were scanned with an energy excitation at between 488 and 535 nm by a confocal laser scanning microscope (Olympus FluoView™ FV1000, Japan). The intensity of green fluorescence photographed was quantified using ImageJ software, as described in Zhang et al. (2019). At least 25 individual roots were used for the quantification of the green fluorescence signal intensity in the N treatment experiment.

### Creation of *NtNRT1.1B*-overexpressing transgenic tobacco lines and quantitative RT-PCR

The *NtNRT1.1B* ORF was amplified by PCR using primers containing the *BamHI* site (5′-TTggatccATGGCACTTCCTGAGACA-3′ and 5′-ATggatccTCAATGACAAAC CGGTCCA-3′) and cloned after the 35S promoter in the vector pCF203. To generate transgenic tobacco plants (with a K326 background) with overexpression of *NtNRT1.1B*, the agrobacterial strain GV3101-mediated leaf-disk method was used, as described above. Several independent homozygous transgenic lines in the T2 generation were obtained on the basis of kanamycin resistance selection and the *NtNRT1.1B* expression test (see **Figure 5A**).

Quantitative reverse transcription (RT)-PCR (qPCR) was performed with total RNA from roots and shoots of both K326 and the transgenic plants created. The procedure for the qPCR experiment followed the same protocol as described in Liu et al. (2018). The relative expression level of *NtNRT1.1B* was normalized to that of two stable internal reference genes (i.e., α-tubulin and α-actin) (Schmidt and Delaney, 2010). qPCR analysis for each line was conducted with four biological replicates, together with three “no template controls” to check the contamination of reagents. Data were analyzed using the 2^−ΔΔCT^ method (CT: cycle threshold) (Schmittgen and Livak, 2008). Before qPCR, amplicons of tested genes were obtained by semiquantitative RT-PCR, gel electrophoresed, purified from the gel, and sequenced for confirmation of their specificity and sequence correctness. The following primers were used: NtNRT1.1B-F, 5′-GGTATCTTTGCCACTGTTCA-3′, and NtNRT1.1B-R, 5′-CAGCATCGTCGA ATTG GTC-3′; Ntα-tubulin-F, 5′-GGTATTCAGGTCGGA AATGCA-3′, and Ntα-tubulin-R, 5′-CTTCGTCAATGACAGTAGGCT- 3′; Ntα-Actin-F, 5′-ATGAGAGAG TGCATATCGATTC-3′ and Ntα-Actin-R, 5′-TTAGTATTCCTCGTTA TCATCGT-3′.

### ^15^N-labeling NO_3_^-1^ root uptake assay

In the experiment of ${\text{NO}}_{3}^{-}$ influx into roots, *NtNRT1.1B*-overexpressing tobacco lines and its corresponding WT (K326) were grown as described above. After 3 weeks’ growth in the normal nutrient solution, plants of a similar size were subjected to N starvation for 2 days, and then roots were exposed for 5 or 30 min to an N-free basic solution supplied with 0.1, 1, or 10 mM total N in the form of ${\text{NO}}_{3}^{-}$ and/or ^15^N- ${\text{NO}}_{3}^{-}$ (in 2.8 L solution; 99.72% ^15^N abundance in K^15^NO_3_, Shanghai Research Institute of Chemical Industry, Shanghai, China). For 10 mM total N supply, only 10% N in the form of ^15^N- ${\text{NO}}_{3}^{-}$ was added to the uptake assay solution; 100% ^15^N-labeled ${\text{NO}}_{3}^{-}$ was used in the 0.1 or 1 mM nitrate supply. After uptake, the roots were washed four times in 1 mM CaSO_4_ for 30 s each, and the roots (after a 5- and 30-min uptake) and shoots (after 30-min uptake) were separately harvested, dried in an oven (at 60°C for 5 days), and ground to a fine powder. Samples (3–4 mg dry matter) were used to determine the ^15^N content in the plants *via* mass spectrometry (DELTA^plus^XP; Thermo-Finnigan, Waltham, MA, USA). The ^15^N incorporated in the roots or shoots was converted into ${\text{NO}}_{3}^{-}$ absorbed by the roots.

For the kinetic study of ${\text{NO}}_{3}^{-}$ transport *via* the NtNRT1.1B pathway, *NtNRT1.1B*-overexpressing tobacco lines (e.g., L1 and L5) and their WT, K326, were used. The roots of 2-day N-starved tobacco plants or those grown with normal nutrient solution after 14-day germination on a 1/2 MS agar plate and 7-day hydroponic culture were exposed for 3 min to the assay solution containing varying ${\text{NO}}_{3}^{-}$ concentrations (in the range of 5,000–7,000 μM; for ${\text{NO}}_{3}^{-}$ concentration > 500 μM or >3,000 μM, 50% or 20% ^15^ ${\text{NO}}_{3}^{-}$ was respectively applied). For the method to determine the ^15^N content, see the above statement. The content of ${\text{NO}}_{3}^{-}$ in *NtNRT1.1B*-overexpressing lines. minus that of the WT plants was used to calculate the absorption by NtNRT1.1B. For details of the protocol for N root uptake analysis, refer to the description by Fan et al. (2017).

### Measurement of nitrate, ammonium, total N, glutamine synthetase, and nitrate reductase

Plant leaves and roots were separately sampled and weighed, and 100 mg of each sample was extracted for the measurement of nitrate, ammonium, glutamine synthetase (GS), and nitrate reductase (NR), following the methods described by Fan et al. (Fan et al., 2017; Liu et al., 2021). The total N per plant (shoot or root) was determined using the Kjeldahl method, as described in Nelson and Sommers (1973).

### Collection of xylem exudate and leaf apoplastic washing fluid

After the 4-week pre-culture of *NtNRT1.1B*-overexpressing tobacco and its WT (K326) with the normal nutrient solution (see above section), plants subjected to N starvation for 2 days or those resupplied with 2 mM ${\text{NO}}_{3}^{-}$ after 2 days’ N starvation were used to sample the xylem (Xy) exudate. The upper part of the plants was removed from the middle of the hypocotyl, and the cutting site of the lower part was immediately wiped three times using sterile water-wetted cotton; the xylem exudate was collected over a 0- to 2-, 2- to 5-, 5- to 8-, 8- to 12-, or 12- to 24-h period after removal of the upper part. The xylem exudate collected in the 0- to 2-h period from the plants grown continuously with the normal nutrient solution (containing 1 mM NH_4_NO_3_ as N source) served as a control sample (CK, **Figure 7B**).

Apoplastic washing fluid (AWF) was extracted from tobacco plants grown for 4 weeks on the normal nutrient solution. AWF was extracted from the upper leaves (UL; i.e., leaves not fully opened), mature leaves (ML; i.e., new fully opened leaves, or the third or fourth leaves down from the UL), and lower leaves (LL; i.e., the sixth and seventh leaves down from the UL). The method for the collection of AWF followed a modified infiltration–centrifugation technique, as described in O'Leary et al. (2014), that is, a vacuum pump instead of a syringe was used for leaf infiltration. The content of ${\text{NO}}_{3}^{-}$ in the xylem exudate and apoplastic fluid was determined using a continuous flow analyzer (AA3, SEAL, Germany).

### Statistical analysis

Descriptive statistics were applied to show the difference in the activity of promoter and enzyme, biomass, the content of total N, ${\text{NO}}_{3}^{-}$ and ${\text{NH}}_{4}^{+}$ , as well as gene expression in plants under varied experimental treatments and/or different tobacco lines. Means and standard deviations (SDs) are plotted in **Figures 4**–**7** and also in **Supplemental Figures 2**, **3**. Statistical analysis was performed using one-way analysis of variance (ANOVA) using the software package SPSS Statistic Client 16.0, SPSS, Beijing, China).

## Results

### Molecular cloning and heterologous expression of *NtNRT1.1B* allowed complementary growth of a yeast nitrate transporter-deletion mutant on nitrate as a sole N source

Being an upland crop, tobacco (*N. tabacum* L.) represents a typical species preferentially using ${\text{NO}}_{3}^{-}$ but not ${\text{NH}}_{4}^{+}$ as an N source for normal growth and development (Fan et al., 2018). Despite physiological evidence showing the existence in tobacco roots of both high- and low-affinity transport systems, which are suggested to contribute to the effective acquisition of N from soils (Fan et al., 2018), descriptions of the molecular basis of ${\text{NO}}_{3}^{-}$ movement in this crop are very limited. To help understand ${\text{NO}}_{3}^{-}$ transport/uptake in/by tobacco at a molecular level, based on our previous, preliminary, study on the genetic information of tobacco *NtNRTs* (Liu et al., 2018), we picked *NtNRT1.2* [cloned first in our previous work (Liu et al., 2018) and renamed as *NtNRT1.1B* (see following section)] as a target to explore its molecular and physiological function in plant nitrate (-N) nutrition. The *NtNRT1.2* gene has been reported to contain a putative open reading frame (with 1,785 bp derived from five exons), which encodes a peptide with 594 amino acid residues and exhibits 12 predicted transmembrane domains (TMDs) with large hydrophilic loops between TMD6 and TMD7 (Liu et al., 2018), similar to *Arabidopsis* AtNRT1.1/1.2.

Recently, based on an updated release of predicted functional gene sequences of tobacco (K326), Zhan et al. (2022) collected 143 putative NPFs and phylogenetically categorized them into eight subfamilies (i.e., NtNPF1–8). However, we have recognized that none of these 143 NtNPFs is identical to the sequence of NtNRT1.1/1.2 described previously by Liu et al. (2018). Thus, we extracted the sequences from the tobacco NtNPF6 subcluster and *Arabidopsis* AtNRT1s/NPF6s and performed a genetic lineage analysis for the NtNR1.1/1.2. The result indicated that both the previously reported NtNRT1.1 and NtNRT1.2 are/share more homologous to AtNRT1.1 than to AtNRT1.2 (AtNPF4.6) (**Supplemental Figure 1**). For this reason, we have renamed NtNRT1.1 and NtNRT1.2 here as NtNRT1.1A and NtNRT1.1B, respectively.

**Figure 1:**
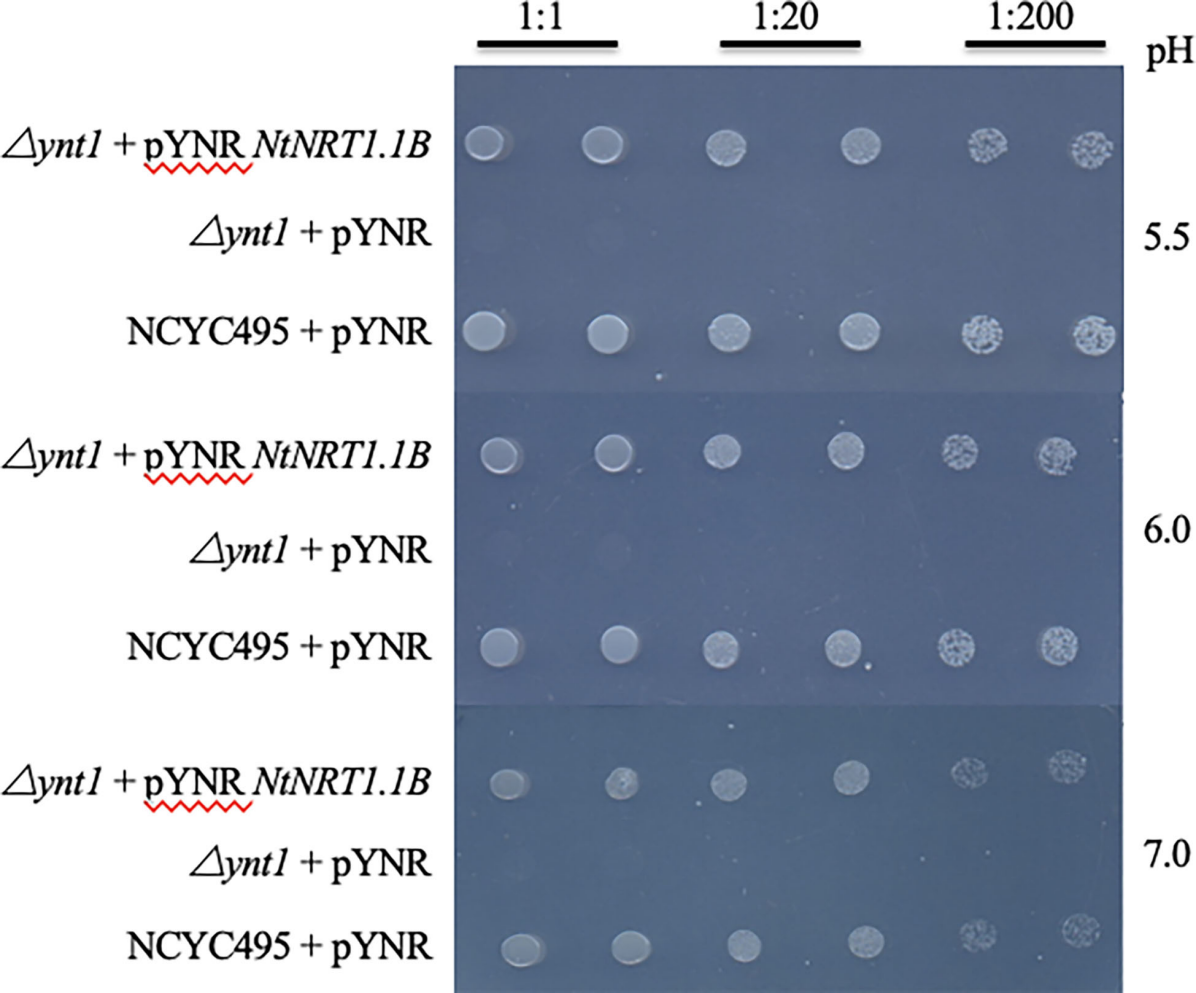
Growth recovery of a nitrate-uptake-defective yeast mutant by heterologous expression of *NtNRT1.1B.* A nitrate transporter-deleted yeast mutant △ynt1 (*△ynt1, △leu*) and its corresponding wild-type strain NCYC495 (*△leu*) were transformed with a yeast expression vector pYNR alone or harboring *NtNRT1.1B*, and then cultured for 3 days first on NAAG medium containing 5 mM ${\text{NO}}_{3}^{-}$ as an N source. A single colony was picked, suspended in 60 μL of sterile water, serially diluted, and dropped (2 μL) onto the NAAG medium containing 0.5 mM ${\text{NO}}_{3}^{-}$ as the sole N source at different pH values (see Materials and methods). An *NtNRT1.1B*-containing △ynt1 mutant showed a functional complemental growth on ${\text{NO}}_{3}^{-}$ , comparable to that of the wild-type NCYC495 (a positive control), whereas △ynt1 harboring the empty vector did not grow (a negative reference). Pictures were taken after 3 days of yeast growth.

To test the functionality of NtNRT1.1B for nitrate transport, a heterologous functional complementation assay in yeast (*H. polymorpha*) was performed. The *NtNRT1.1B* ORF was cloned into the yeast expression vector pYNR and transformed into the nitrate transporter-disrupted yeast mutant △ynt (△*ynt*, △*leu*) (see Materials and methods), which is unable to grow on less than 0.5 mM ${\text{NO}}_{3}^{-}$ as a sole N source (Pérez et al., 1997). The result showed that compared with transformants carrying the empty vector pYNR, yeast cells of △ynt harboring *NtNRT1.1B* restored the growth on 0.5 mM ${\text{NO}}_{3}^{-}$ , comparable to its WT strain, NCYC495 (△*leu*), transformed with pYNR as a positive control (**Figure 1**). In addition, as some nitrate and peptide transporters characterized in the NRT1/PTR family are reported to be proton-coupled permeases for the movement of nitrate, potassium, and/or auxin (Wang et al., 2012; Watanabe et al., 2020), the effect of medium pH on *NtNRT1.1B*-facilitated ${\text{NO}}_{3}^{-}$ transport in yeast was examined. However, pYNR-*NtNRT1.1B*-harboring transformants showed no obvious difference in growth rate at pH levels ranging from 5 to 7 (**Figure 1**), suggesting that the *NtNRT1.1B*-encoding protein could mediate ${\text{NO}}_{3}^{-}$ import from media across the plasma membrane (PM) of yeast cells independent of pH.

**Figure 2:**
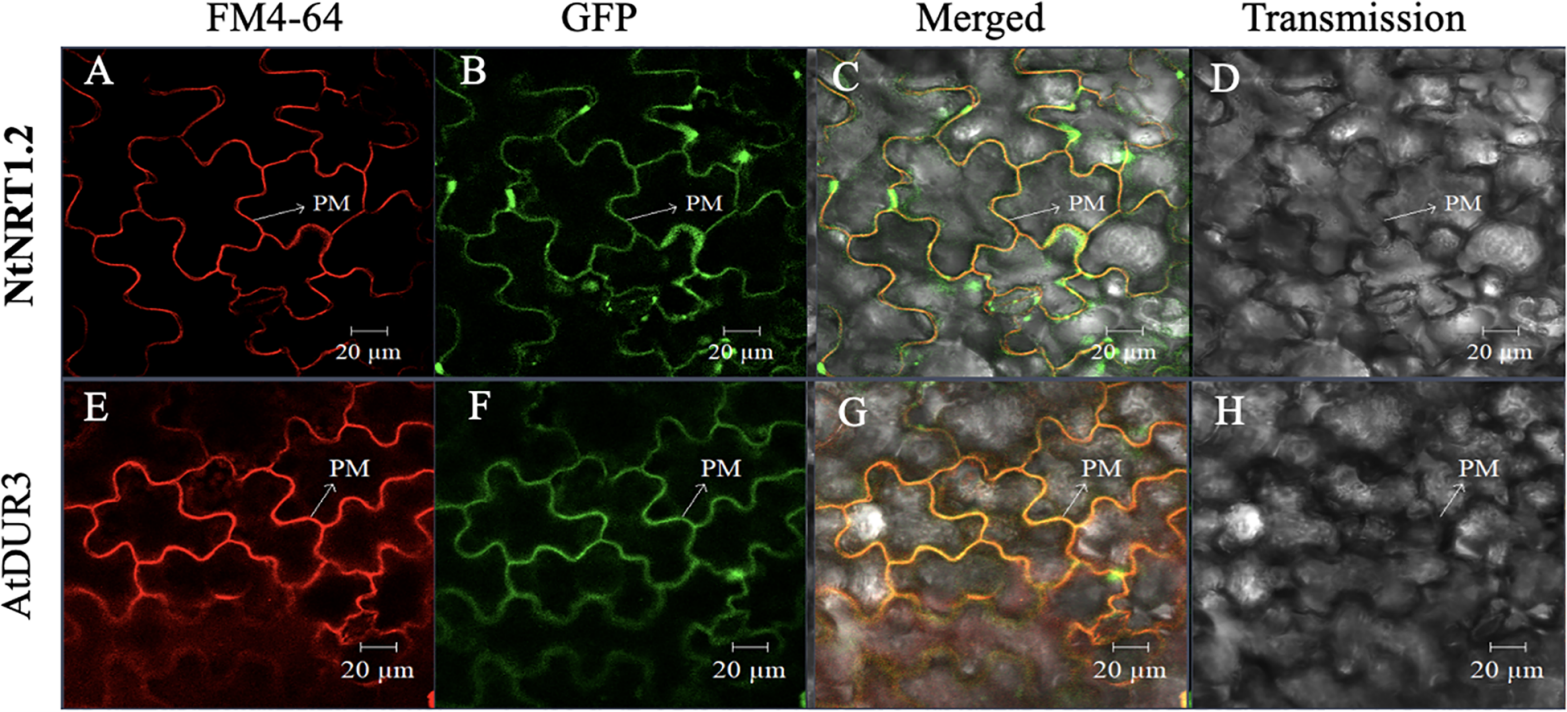
Subcellular localization of green fluorescent protein (GFP)-tagged *NtNRT1.1B* in tobacco leaf epidermal cells. *Agrobacterium* GV3101 harboring the *NtNRT1.1B::GFP* construct was infiltrated into the epidermal cells of tobacco leaves for a transient expression of *NtNRT1.1B*, which was fused to the *GFP* gene (see Materials and methods). Representative images of the leaf epidermal cells were visualized using laser scanning confocal microscopy (see Materials and methods). The plasma membrane (PM) was dyed red with a chemical marker, FM 4-64, for 10 min (Bolte et al., 2004). The red **(A, E)**, green **(B, F)**, and yellow **(C, G)** are derived from the PM-staining marker FM 4-64, GFP, and superposition of red and green, respectively. **(D, H)**, images of leaf epidermal cells visualized in a transmission pattern. AtDUR3 (an *Arabidopsis* PM-localized high-affinity urea transporter; Liu et al., 2003) was used as a reference. FM 4-64, *N*-(3-triethylammoniumpropyl)-4-(p-diethylaminophenyl-hexatrienyl) pyridinium dibromide; PM, plasma membrane. Scale bars: 20 μm.

Furthermore, to survey the possibility of nitrate permeation through the cell membrane(s) *via* the NtNRT1.1B pathway, subcellular localization of NtNRT1.1B was determined using a protein fusion approach, in which a GFP gene (*GFP*) was C-terminally fused with the *NtNRT1.1B* ORF to yield *NtNRT1.1B::GFP*, which was constructed downstream of the CaMV 35S promoter (see Materials and methods). After transient expression of *NtNRT1.1B::GFP* in tobacco leaf epidermal cells, the GFP signal in the cells was visualized using confocal microscopy (see Materials and methods). NtNRT1.1B::GFP resulted in a green signal, in a fine-line pattern (**Figure 2B**), which overlapped substantially with the red fluorescence of the PM (**Figure 2A**) indicated by the signal derived from a 10-min incubation of the cells with the chemical FM 4-64 (Bolte et al., 2004). This overlap was clearly seen in the yellow of the superposition image that was the result of the merged green and red signals (**Figure 2C**), very similar to that of *Arabidopsis* high-affinity urea transporter AtDUR3 (**Figures 2E–G**), which is documented to be targeted at the PM (Liu et al., 2003). Thus, the subcellular localization of NtNRT1.1B should mainly reside on the PM of tobacco cells.

### Promoter activity of *NtNRT1.1B* is mainly detected in vascular and reproductive tissues and is regulated by nitrogen status in roots

**Figure 3:**
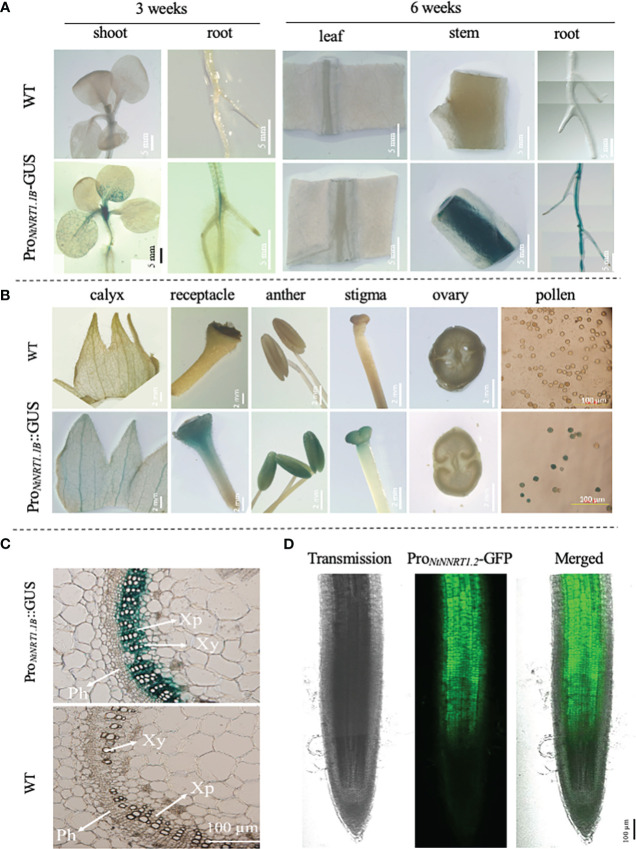
Analysis of promoter activity of the *NtNRT1.1B* gene in tobacco lines transformed with Pro*_NtNRT1.1B_*-β-glucuronidase (*GUS*) or -green fluorescence protein (GFP). A genomic sequence 2,000 bp upstream from a putative start codon of *NtNRT1.1B* was cloned before the *GUS* or *GFP* marker gene, and then transformed into tobacco plants (K326) to create different transgenic lines generated for promoter activity assay under normal or confocal microscopy (see Materials and methods). **(A, B)** Observation of GUS activity in the various tissues of the Pro*_NtNRT1.1B_*-*GUS* transgenic tobacco during a vegetative stage, i.e., 3 or 6 weeks’ growth **(A)** and reproductive stage **(B)**. **(C)** Detection of GUS activity in a cross-section of stems of 6-week-old tobacco plants. Plants were grown hydroponically with 1 mM NH_4_NO_3_ as the N source. Similar expression patterns of GUS driven by Pro*_NtNRT1.1B_* were observed in three independent transgenic lines. **(D)** Occurrence of GFP actuated by Pro*_NtNRT1.1B_* in the primary root stele. Six-week-old transgenic tobacco plants grown hydroponically with 2 mM NH_4_NO_3_ were used. WT, wild type; Xy, xylem; Ph, phloem; Xp, xylem parenchymal cells. Scale bars are indicated in pictures.

The spatiotemporal transcription of a functional gene is an important issue that may reflect its potential biological role in plant growth and development. To illustrate the expression patterns of *NtNRT1.1B* with regard to tissue/organ specificity and N nutritional response, a promoter activity assay using a GUS/GFP reporter approach was conducted. A 2,000-bp upstream sequence (Pro*_NtNRT1.1B_*) of *NtNRT1.1B* ORF was cloned directly before the *GUS* or *GFP* gene, and then transformed into tobacco (K326; see Materials and methods). The reporter expression reflecting promoter activity was visualized and/or quantified by (confocal) microscopic analysis (note that for the histochemical detection of GUS, plant tissues were subjected to GUS staining; see Materials and methods) (**Figure 3**). The blue color derived from the Pro*_NtNRT1.1B_*-tiggered GUS expression occurred clearly in the area of the young (3-week) shoot apex and lateral root primordia, and in the 6-week plant (primary) root and shoot–stem tissue, as well as flower tissues including the receptacle, stigma, anther, and pollen grains (**Figures 3A, B**). A close-up visualization of a cross-section of GUS-stained tissue further revealed a strong appearance of the Pro*_NtNRT1.1B_* activity in the xylem parenchymal cells of the stem vascular stele (**Figure 3C**), and this expression pattern is comparable to an observation of Pro*_NtNRT1.1B_* driven GFP occurrence in the root, where green fluorescence signals were strongly detected in the stele area of the mature zone of the primary root (**Figure 3D**).

**Figure 4:**
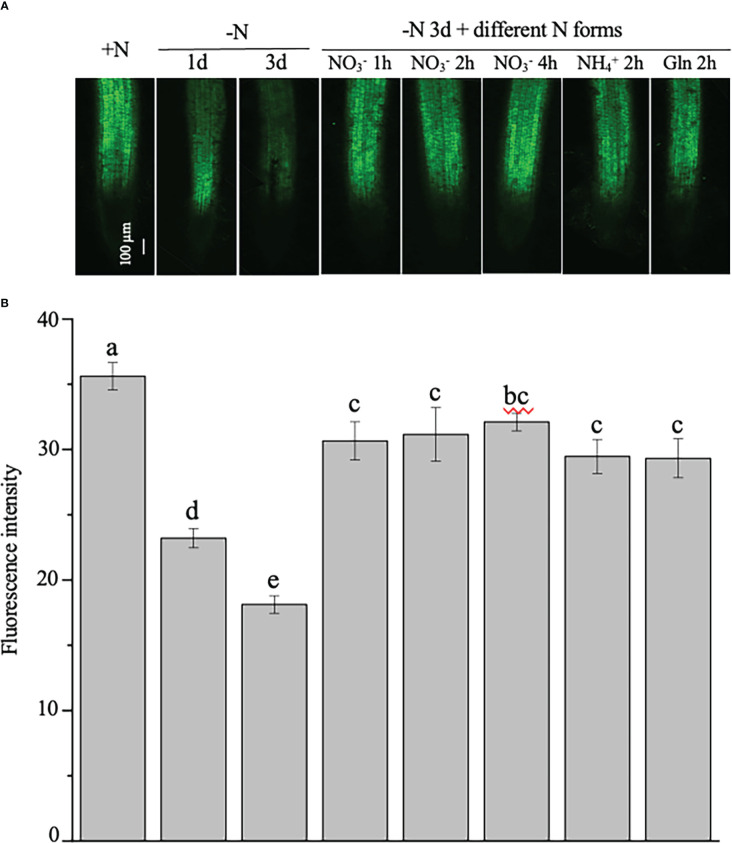
Examination of the regulation of the expression of the green fluorescence protein (GFP) indicated Pro*_NtNRT1.1B_* activity by nitrogen in tobacco roots. Transgenic plants expressing *GFP* driven by a putative *NtNRT1.1B* gene promoter (Pro*_NtNRT1.1B_*) were grown hydroponically with 1 mM NH_4_NO_3_ as a nitrogen source for 4 weeks (see Materials and methods), and then were starved of N for 1 or 3 days. Furthermore, those plants subjected to 3 days’ N starvation were resupplied with different N (2 mM) forms for different time periods. **(A)** Representative images of the GFP signal in roots. +N, continuous nitrogen-supply; –N, nitrogen starvation; Gln, glutamine. Confocal microscopic analysis is described in Materials and methods. Bar = 100 μm. **(B)** Quantification of green fluorescence intensity in roots of plants treated with nitrogen regimes. The intensity of the green fluorescence was photographed was then quantified using ImageJ software (see Materials and methods). The means of 15 biological replicates ± standard deviation (SD) (*n* = 15 individual root samples) were plotted and different letters above the bars indicate statistically significant differences [*P<* 0.05 by one-way analysis of variance (ANOVA) and a multiple comparison test].

To test the stability of the *NtNRT1.1B* promoter activity in the root, GFP signals were further quantitatively analyzed in the roots of 4-week-old tobacco plants, which were N starved for 1 or 3 days, with N resupplied (in the form of ${\text{NO}}_{3}^{-}$ , ${\text{NH}}_{4}^{+}$ , or Gln) to the 3-day N-starved plants for 1, 2, or 4 h (see Materials and methods) before measurement. As shown in **Figure 4A**, the green fluorescence intensity was strongly reduced by medium N depletion (e.g., for 1 or 3 days) in the roots compared with that in the control grown with continuous N provision. Resupply (for 1–4 h) of different N sources, including *NtNRT1.1B*’s putative substrate, ${\text{NO}}_{3}^{-}$ , to the 3-day N-starved tobacco rapidly and markedly enhanced GFP accumulation in the roots relative to that of the control (i.e., the 3-day N-starved tobacco; **Figure 4B**). Such N-status-regulated GFP expression patterns triggered by Pro*_NtNNRT1.1B_* activity were confined only in a comparable root tissue area, namely the root stele in the mature zone (**Figure 4A**).

### Effect of *NtNRT1.1B* overexpression on tobacco growth and nitrate transport

**Figure 5:**
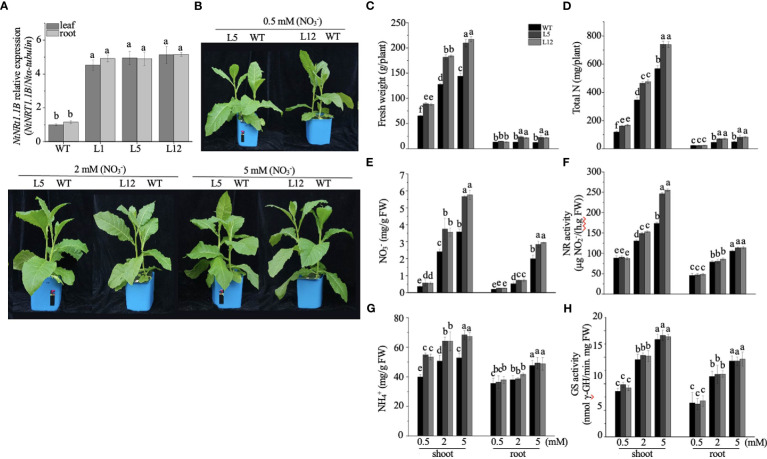
Overexpression of *NtNRT1.1B* significantly enhances the growth of tobacco plants. *NtNRT1.1B*-transformed tobacco lines and their WT (K326) plants were hydroponically cultured for 25 days with a supply of ${\text{NO}}_{3}^{-}$ as the sole N source at a concentration of 0.5, 2, or 5 mM (see Materials and methods). For the measurement of N-related physiological components (see Materials and methods), the second and third fully expanded leaves counted down from the top were sampled; four biological replicates were conducted. **(A)** Detection of *NtNRT1.1B* transcript abundance. Roots and shoots of three independent *NtNRT1.1B*-overexpressing lines (L1, L5, and L12) and WT grown on 1/2 Murashige & Skoog (MS) agar plates for 14 days were sampled for gene expression by using qPCR (see Materials and methods). The expression level of *NtNRT1.1B* relative to that of α-tubulin (set to 1) was calculated and plotted. Data were obtained from four biological replicates. **(B)** Images of representative growth phenotype of *NtNRT1.1B*-overexpressing lines and WT tobacco grown with ${\text{NO}}_{3}^{-}$ as the sole N source. Plant culture is as described above. **(C–H)** Quantification of the fresh weight of shoots and roots **(C)**, total N **(D)**, the content of ${\text{NO}}_{3}^{-}$ **(E)** and ${\text{NH}}_{4}^{+}$ **(G)**, and enzymatic activity of glutamine synthetase GS **(F)** and nitrate reductase NR **(H)**. Means ± standard deviation (SD) (*n* = 4) are depicted, and different letters indicate statistically significant differences [*P*< 0.05, one-way analysis of variance (ANOVA)]. Similar results were obtained from experiments with plants cultured under in pots with soil growth conditions (see **Supplemental Figure 1**).

To appreciate the potential biological role of *NtNRT1.1B* in its native plant, an expression construct of *NtNRT1.1B* ORF driven by the CaMV 35S promoter was cloned and subsequently introduced into tobacco K326 (WT); three independent *NtNRT1.1B* overexpression (*OE-NtNRT1.1B*) homozygous lines were generated for the physiological study (see Materials and methods). Gene expression measurements showed that, in both roots and shoots/leaves, around fourfold higher mRNA abundance of *NtNRT1.1B* relative to that in the WT was confirmed by quantitative RT-PCR (**Figure 5A**).

For the growth phenotyping and N nutritional physiological assay, transgenic and WT tobacco plants were cultivated hydroponically with nitrate as a sole N source at different concentrations (0.5, 2.0, and 5.0 mM) for 25 days (see Materials and methods). Compared with the WT, *OE-NtNRT1.1B* lines were bigger and exhibited greater biomass production, particularly for shoots with 35%–46% higher upper part fresh weight under 0.5–5 nitrate provision (**Figures 5B, C**). Accordingly, the content of total N, ${\text{NO}}_{3}^{-}$ , and ${\text{NH}}_{4}^{+}$ in the shoots of *OE-NtNRT1.1B* plants was measured to be markedly higher than that of the control (**Figures 5D, E, G**). A similar tendency was also observed in the roots of the transgenic lines relative to the WT (**Figures 5C–E**), with the exception that root ${\text{NH}}_{4}^{+}$ content was not significantly different between the WT and *OE-NtNRT1.1B* lines (**Figure 5G**). Metabolically, measuring the activity of two enzymes required for the first step of ${\text{NO}}_{3}^{-}$ or ${\text{NH}}_{4}^{+}$ assimilation revealed that NR action in the shoots was proportionally increased at elevated nitrate levels (e.g., ≈ 2–5 mM), and in particular was 20%–41% higher in the *NtTR1.2*-transgenic plants than in the WT (**Figure 5F**). However, the activity of NR in the roots and of GS in both shoots and roots remained comparably stable in both the transgenic and WT plants (**Figures 5F, H**). An increase in the activity of NR and GS in both roots and shoots with elevated medium N concentrations (0.5–5 mM) agrees with the observation reported by Fan et al. (2018). When plants were grown in pots with soil supplied with nitrate at four rates (0, 0.5, 2, and 5 mM; **Supplemental Figure 2A**), results very similar to those achieved by hydroponics, described above, were observed (**Supplemental Figure 2**).

**Figure 6:**
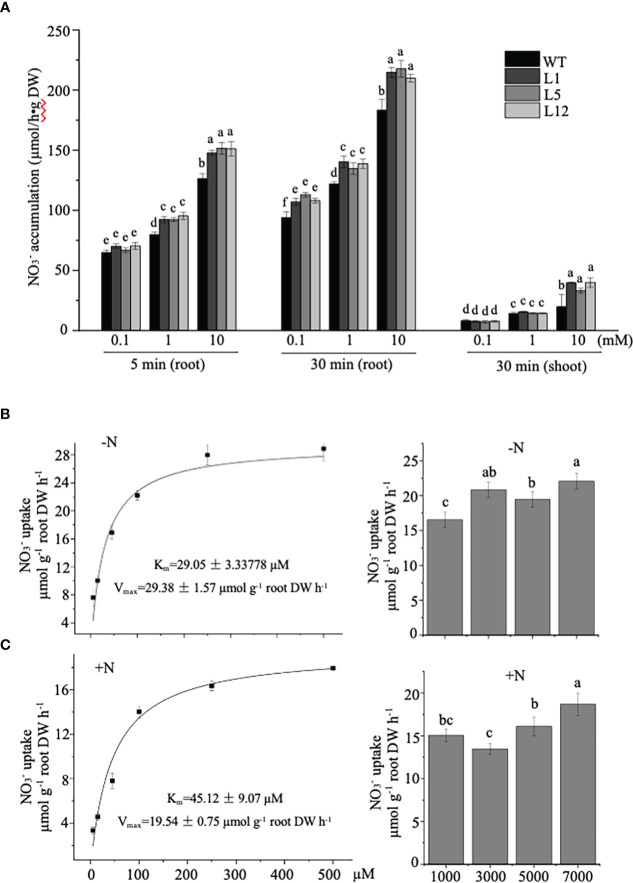
Measurement of nitrate influx into roots of *NtNRT1.1B*-expressing lines and their WT tobacco. **(A)** Time-dependent ^15^N-labeling ${\text{NO}}_{3}^{-}$ absorption by roots of *NtNRT1.1B*-expressing lines and their WT. ^15^N- ${\text{NO}}_{3}^{-}$ uptake by roots was conducted with *NtNRT1.1B*-overexpressing lines (L1, L5, and L12) and their WT (K326) cultivated hydroponically for 4 weeks and then starved of N for 2 days. The exposure of the roots for 5 min or 30 min in an N-free nutrient solution supplied with ${\text{NO}}_{3}^{-}$ at three concentrations (0.1, 1.0, or 10 mM) was designed for the influx measurement. A detailed protocol for the tobacco growth and root uptake study is provided in Materials and methods. Tracer ^15^N- ${\text{NO}}_{3}^{-}$ with 99.72% ^15^N abundance (K^15^NO_3_) was applied (note that, for the 10 mM ${\text{NO}}_{3}^{-}$ supply, only 10% N in the form of ^15^ ${\text{NO}}_{3}^{-}$ was added to the uptake assay solution). The ^15^N incorporated into plant samples (3–4 mg roots or shoots) was analyzed by using mass spectrometry (see Materials and methods) and was converted into ${\text{NO}}_{3}^{-}$ absorbed by the roots. The mean values ± standard deviation (SD) (*n* = 4 biological replicates) are shown, and statistically significant differences are indicated by different letters [*P*< 0.05 by one-way analysis of variance (ANOVA)]. DW, dry weight. **(B, C)** Concentration-dependent short-term influx of ^15^ ${\text{NO}}_{3}^{-}$ into roots of *NtNRT1.1B*-expressing line and its WT. After germination of seeds on 1/2 MS agar plates for 14 days, seedlings were transferred to a normal nutrient solution for 7 days of growth. Roots of 2-day N-starved plants or those grown with the normal nutrient solution were used in the influx assay; the roots were supplied with 5,000–7,000 μM ${\text{NO}}_{3}^{-}$ (in the form of 100% ^15^ ${\text{NO}}_{3}^{-}$ or partially containing ^15^ ${\text{NO}}_{3}^{-}$ ; see Materials and methods) for 3 min (to minimize the long-distance movement of nitrate absorbed). Values are differences in ^15^ ${\text{NO}}_{3}^{-}$ accumulation in the WT transformed with or without *NtNRT1.1B* (the *NtNRT1.1B*-overexpressing line1/L1 was used). The influx of nitrate into roots *via* NtNRT1.1B was saturable and displayed Michaelis-Menten kinetics with a half-maximal saturation at around 30-45 µM nitrate (i.e. an affinity constant Km of NtNRT1.1B for nitrate). The mean values ± SE (*n* = 5 or 6) are shown, and different letters above the bars indicate statistically significant differences [(*P*< 0.05 by one-way analysis of variance (ANOVA)]. ^15^N incorporated in the roots was analyzed by using mass spectrometry and converted into ${\text{NO}}_{3}^{-}$ taken up by the roots. “–N”, 2-day N-starved plants; “+N”, plants grown continuously with normal nutrient solution. A very similar result was also observed in *NtNRT1.1B*-transgenic line 5 (L5; see **Supplemental Figure 3**).

To determine if the growth improvement in transgenic tobacco was directly linked to ${\text{NO}}_{3}^{-}$ uptake/transport strengthened by *NtNRT1.1B* overexpression, we conducted a short-term root influx study using ^15^N- ${\text{NO}}_{3}^{-}$ tracer under a nitrate supply at three 10-fold differing levels (**Figure 6A**; see Materials and methods). The measurement of ^15^N revealed that the *NtNRT1.1B*-transgenic tobacco roots could accumulate 15%–20% more ${\text{NO}}_{3}^{-}$ (by exposure for 5 min or 30 min of roots to a ${\text{NO}}_{3}^{-}$ containing solution) than the WT (**Figure 6A**); approximately 50% more ${\text{NO}}_{3}^{-}$ was deposited in the shoots of the transgenic lines than in the shoots of the WT after a 30-min root exposure to 10 mM ${\text{NO}}_{3}^{-}$ (**Figure 6A**), although overall ${\text{NO}}_{3}^{-}$ accumulation in the shoots was lower than in the roots during this short-term ${\text{NO}}_{3}^{-}$ supplementation. Thus, an increase in *NtNRT1.1B* expression in a constitutive pattern did enhance the root acquisition of ${\text{NO}}_{3}^{-}$ from external media.

To further assess a transport property of NtNRT1.1B for ${\text{NO}}_{3}^{-}$ across the plant cell membrane, short-term influx into the roots of 7-day hydroponically cultivated *NtNRT1.1B*-overexpressing plants (line 1) and their WT with or without 2 days’ N starvation, was measured after a 3-min exposure of the roots to the ^15^ ${\text{NO}}_{3}^{-}$ containing nutrient solution [see Materials and methods; note: by 3 min uptake, an increase in^15^N in shoots was not detectable, indicating no movement of root-absorbed ^15^N to the upper parts of the plant (data not shown)]. At a range of sub-millimolar concentrations (5–500 µM tested), ${\text{NO}}_{3}^{-}$ root influx showed that NtNRT1.1B did facilitate an import of ${\text{NO}}_{3}^{-}$ into the roots, and followed Michaelis–Menten kinetics (**Figure 6B**). The root absorption *via* NtNRT1.1B was saturated at ≈ 500 μM ${\text{NO}}_{3}^{-}$ , and exhibited maximal transport activity, i.e., *V*_max_, at 29.38 ± 1.57 or 19.54 ± 0.75 μmol ${\text{NO}}_{3}^{-}$ g^–1^ dry weight (DW) h^–1^ as well as a transport affinity constant, *K*_m_, at 29.05 ± 3.34 or 45.12 ± 9.07 μM for ${\text{NO}}_{3}^{-}$ under root N starvation or N provision (**Figure 6B**), respectively. This kinetic property of NtNRT1.1B for ${\text{NO}}_{3}^{-}$ transport was very similarly detected in another transgenic line (**Supplemental Figure 3**). Although the measured values of *K*_m_ and *V*_max_ of NtNRT1.1B for ${\text{NO}}_{3}^{-}$ are numerically different on N depletion or N supply in the growth medium, which may affect plant internal N status and/or metabolism, such *K*_m_ or *V*_max_ data fall into a similar value range. At higher ${\text{NO}}_{3}^{-}$ concentrations (i.e., 1,000–7,000 µM), root ${\text{NO}}_{3}^{-}$ accumulation values derived by subtracting the value in *NtNRT1.1B*-overexpressing plants from that in WT plants at the same external ${\text{NO}}_{3}^{-}$ concentration could not fit any possible mechanism equation (**Figure 6B**). Thus, NtNRT1.1B could be considered as a high-affinity component for nitrate permease in tobacco plants.

### Effect of *NtNRT1.1B* overexpression on internal translocation of NO_3_^-^ in tobacco

**Figure 7:**
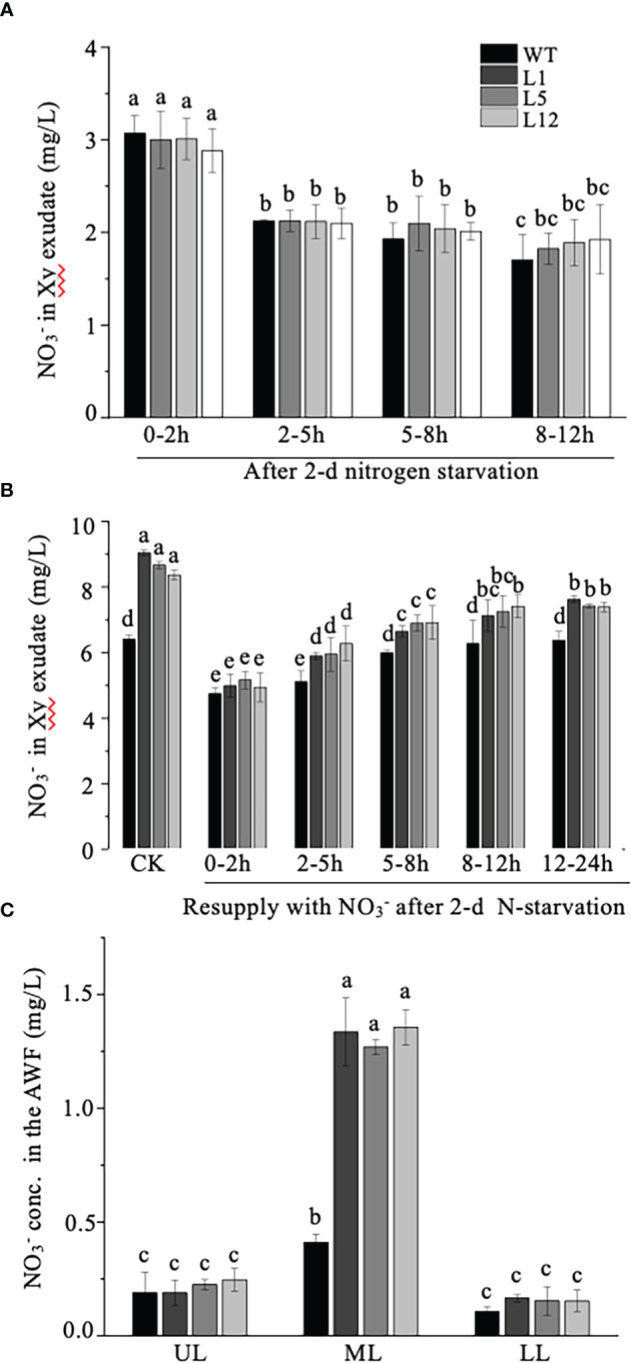
Determination of ${\text{NO}}_{3}^{-}$ concentration in xylem exudate and apoplastic washing fluid (AWF) of *NtNRT1.1B*-overexpressing plants and WT tobacco. **(A, B)** ${\text{NO}}_{3}^{-}$ concentration in the xylem exudate. After 4 weeks of hydroponic pre-culture of *NtNRT1.1B*-overexpressing tobacco lines and their WT (K326) with the normal nutrient solution, plants were subjected to N starvation for 2 days or those resupplied with 2 mM ${\text{NO}}_{3}^{-}$ after 2 days’ N starvation were used to collect xylem exudate (see Materials and methods). The xylem exudate derived from the plants starved of N **(A)** or resupplied with ${\text{NO}}_{3}^{-}$ after 2 days’ N starvation **(B)**. Samples were collected over a 0- to 2-, 2- to 5-, 5- to 8-, 8- to 12-, or 12- to 24-h period after removal of the plant’s upper parts. Xy, xylem. CK, the sample collected from the plants grown with 1 mM NH_4_NO_3_ as the N source and served as the control. **(C)** ${\text{NO}}_{3}^{-}$ concentration of AWF. The AWF was extracted from different leaves of the plants grown for 4 weeks under hydroponic culture conditions (see Materials and methods). UL, ML, and LL indicate upper, middle, and lower leaf, respectively (see Materials and methods). The concentration of ${\text{NO}}_{3}^{-}$ was determined using a flow analyzer (see Materials and methods). At least six biological replicates were conducted. The means + standard deviation (SD) (*n* = 6) were plotted, and different letters indicate statistically significant differences [*P*< 0.05, one-way analysis of variance (ANOVA)].

As *NtNRT1.1B* represents a homolog of nitrate transporters and its promoter activity could be clearly detected in the stem xylem area and root stele (**Figures 3C, D**), we speculated that NtNRT1.1B might favor nitrate translocation from the roots to the upper parts of the plant. To test this hypothesis, the content of ${\text{NO}}_{3}^{-}$ in the root xylem exudate and leaf AWF was measured. Four-week-old plants starved of N for 2 days or resupplied with 3 mM ${\text{NO}}_{3}^{-}$ after 2 days’ N starvation were excised at the stem 2–3 cm above the root base for xylem exudate collection, and differently aged leaves were sampled for AWF extraction (see Materials and methods). Under 2-days’ N deprivation, ${\text{NO}}_{3}^{-}$ in the root exudate was detectable with gradually reduced concentrations over the period of a continuous xylem sap collection (0–12 h), but there was no significant difference between WT and transgenic plants (**Figure 7A**). Following resupplementation of ${\text{NO}}_{3}^{-}$ to the 2-day N-starved roots, ${\text{NO}}_{3}^{-}$ concentration in the root exudate was 15%–20% higher in the *OE-NtNRT1.1B* lines than in the WT (except for the sample collected in the first 2 hours) (**Figure 7B**); this *NtNRT1.1B* overexpression-related elevation of ${\text{NO}}_{3}^{-}$ in xylem saps was also clearly observed in the control treatment (CK, the plants grown with the normal nutrient solution containing 1 mM NH_4_NO_3_) (**Figure 7B**). Furthermore, preparation of the leaf AWF allowed us to determine a generally increased concentration of ${\text{NO}}_{3}^{-}$ in the out-cell-space fluid of mature leaves (**Figure 7C**), in which *NtNRT1.1B*-transformed lines even showed a threefold higher concentration of ${\text{NO}}_{3}^{-}$ in their AWF than the WT (**Figure 7C**). These data may indicate at least a significant effect or role for NtNRT1.1B in facilitating the long-distance translocation of nitrate between the roots and the upper parts of plants.

## Discussion

Nitrate serves as a principal N source from soils for plant growth and also as an intriguing signal molecule that regulates many biological processes, including gene expression, root system architecture (Vidal et al., 2020), leaf development (Rahayu et al., 2005), seed dormancy (Alboresi et al., 2005), and flowering time (Castro et al., 2011). To date, the uptake and internal translocation as well as sensing of ${\text{NO}}_{3}^{-}$ have been mostly attributed to the molecular action of the NRT1 and NRT2 family of proteins *in planta* (Carillo and Rouphael, 2022; Gao et al., 2022). Despite the elaborate functional characterization of some NRT1s and NRT2s in certain plant species, including *Arabidopsis*, rice, and soya beans (Vidal et al., 2020; Carillo and Rouphael, 2022), no individual molecular component responsible for ${\text{NO}}_{3}^{-}$ movement in cultivated tobacco has been described.

Based on our previous work showing putative coding sequences of *N. tabacum* nitrate permease homologs *NtNRTs* with their tissue-specific and N nutritional-related expression patterns (Liu et al., 2018), here we functionally analyzed *NtNRT1.1B* (i.e., what was previously termed *NtNRT1.2* in Liu et al., 2018; **Supplemental Figure 1**) for its significance in plant growth associated with nitrate transport and N nutrition. We have provided experimental evidence that a putative ORF of *NtNRT1.1B* of 1,785 bp in length, which encodes 594 amino acid residues, exhibits a function in mediating ${\text{NO}}_{3}^{-}$ permeation into cells. First, heterologous expression of *NtNRT1.1B* could restore the growth of an ${\text{NO}}_{3}^{-}$ uptake-defective yeast mutant, *△ynt1 (H. polymorpha*), on 0.5 mM ${\text{NO}}_{3}^{-}$ as its sole N source, suggesting a possible molecular function of NtNRT1.1B in ${\text{NO}}_{3}^{-}$ import into yeast cells (**Figure 1**). This is similar to the case of many other identified N source transporters, such as NtAMTs for ammonium and OsDUR3 for urea (Liu et al., 2003; Liu et al., 2015; Fan et al., 2017), whose heterologous expression enables a functional complementation of a related yeast mutant on a given selective growth medium. Second, a protein subcellular localization of NtNRT1.1B indicated by a transient expression of *NtNRT1.1B::GFP* in tobacco leaf epidermal cells (**Figure 2**) points to a great possibility of ${\text{NO}}_{3}^{-}$ permeation across the plasma membrane *via* the NtNRT1.1B pathway. Third, constitutive overexpression of *NtNRT1.1B* in its native plants grown with ${\text{NO}}_{3}^{-}$ as their sole form of N could remarkably enhance the accumulation in shoot and/or root of total N, ${\text{NO}}_{3}^{-}$ , and even ${\text{NH}}_{4}^{+}$ as compared with that in WT (**Figures 5D, E, G**), resulting in significant improvement in the growth of transgenic lines, and higher biomass production, at least at a vegetative stage and when tested under hydroponics (**Figures 5A–C**). This NtNRT1.1B-mediated increase in nitrogen (in the form of ${\text{NO}}_{3}^{-}$ ) acquisition/accumulation in tobacco was further supported by a short-term root influx study, which showed a 15%–20% greater ${\text{NO}}_{3}^{-}$ deposition in the *NtNRT1.1B* overexpressors (**Figure 6A**), as well as a high affinity of NtNRT1.1B for ${\text{NO}}_{3}^{-}$ at a *K*_m_ of 30–45 µM (**Figure 6B**). In addition, GFP expression-indicated promoter action assay clearly showed that the activity in roots of 2000bp upstream of *NtNRT1.1B* ORF did respond to plant internal N-nutritional status and external presence of different N forms, including its putative substrate ${\text{NO}}_{3}^{-}$ (**Figure 4A**), namely a down-regulation of the promoter activity by N-starvation and induction by N-resupply after 3d N-depletion (**Figures 4A, B**). This pattern is similar to the previously published results when *NtNRT1.1B* mRNA abundance was measured (Liu et al., 2018). Such resulting data may rationally suggest that our identified *NtNRT1.1B* coding sequence should have at least a role in ${\text{NO}}_{3}^{-}$ transport associated with the plant’s effective use of ${\text{NO}}_{3}^{-}-\text{N}$ nutrition, thus adding a new functional homologous member to the plant NRT1 family as a nitrate transporter.

Tissue-/organ-specific expression patterns of a functional gene may provide a valuable clue for proposing its molecular function(s) in plant growth and development. *NtNRT1.1B* expression, indicated by its putative promoter activity, was detected in the stele region of the more mature parts of roots (**Figure 3D**), xylem parenchymal cells of the stem vascular stele (**Figure 3C**), flower tissues and pollen grains (**Figure 3B**), and in the root–shoot junction region (**Figure 3A**). Together with measurements of higher nitrate concentrations in xylem exudate and AWF of *NtNRT1.1B*-overexpressing lines, this finding suggests that NtNRT1.1B plays a role in the long-distance transport (*via* xylem loading) of ${\text{NO}}_{3}^{-}$ and its translocation to and distribution among the upper parts of plants, whenever required, rather than in the uptake of ${\text{NO}}_{3}^{-}$ from external environments.

A crucial characteristic of a transporter is its specificity and affinity to a substrate(s). Being a member of the NRT1/PRT family (NPF), it is documented that both *Arabidopsis* CHL1/AtNRT1.1/NPF6.3 and AtNRT1.2/NPF4.6 are low-affinity transporters with a similar *K*_m_ of around 5.9–8.5 mM for ${\text{NO}}_{3}^{-}$ when measured in a heterologous system *Xenopus* oocytes [Huang et al., 1996; Huang et al., 1999; but AtNRT1.1 is actually a dual-affinity nitrate transporter (Liu et al., 1999)], mainly contributing to root uptake of ${\text{NO}}_{3}^{-}$ *via* low-affinity transport systems (Carillo and Rouphael, 2022). Although NtNRT1.1 and NtNRT1.2 (renamed in this work as NtNRT1.1A and NtNRT1.1B; **Supplemental Figure 1**) had been identified in a previous publication (Liu et al., 2018), they seem to be CHL1/AtNRT1.1 duplicates in tobacco, because NtNRT1.1A and NtNRT1.1B share 67.8% and 67.1% identity, respectively, with AtNRT1.1 and 87.3% identity with each other, and share only 38% and 36.6% homology with AtNRT1.2, respectively (Liu et al., 2018). This is similar to tomato LeNRT1-1 and -2 to AtNRT1.1 (Lauter et al., 1996). Nevertheless, NtNRT1.1B, being an ortholog of NRT1, could transport nitrate as one of its native substrates with great potential, and this can be experimentally strengthened by our observations of yeast functional complementation (**Figure 1**), an enhanced accumulation of total N, ${\text{NO}}_{3}^{-}$ , and ${\text{NH}}_{4}^{+}$ in its overexpressing lines when grown in or supplied with nitrate (**Figures 5D, E, G**, **6A**), and a putative transport affinity constant of NtNRT1.1B for ${\text{NO}}_{3}^{-}$ at about 30–45 µM (**Figure 6B**). Regarding other possible substrates for NtNRT1.1B, based on previous publications showing molecular actions of NRT1s from other plant species (for a recent review see Carillo and Rouphael, 2022), it should be interesting and necessary to analyze whether or not NtNRT1.1B would also permeate Cl^–^, auxin, or ABA, etc., which have been shown to be transported by *Arabidopsis* AtNRT1.1 (transporting chloride and auxin as well) or AtNRT1.2 (also permeating ABA) (Huang et al., 1999; Liu et al., 1999; Kanno et al., 2012; Zhang et al., 2021).

In conclusion, the present work provides molecular and physiological evidence that tobacco *NtNRT1.1B* with a 1,785-bp coding sequence may function as an effective genetic component responsible for ${\text{NO}}_{3}^{-}$ transport in plants. Based on our observations of the growth complementation on ${\text{NO}}_{3}^{-}$ of a yeast mutant by *NtNRT1.1B*, its promoter activity was mainly in root and shoot vascular tissues, with plasma membrane protein localization, .. root influx, and higher ${\text{NO}}_{3}^{-}$ content in xylem sap and AWF of *NtNRT1.1B*-overexpressing tobacco, NtNRT1.1B should be considered as a functional permease for ${\text{NO}}_{3}^{-}$ across the PM and transporting across the plasma membrane and translocating from the roots to the shoots. Most significantly, *NtNRT1.1B* overexpression in its native plants obviously enhanced the growth of tobacco grown on nitrate as an N source, suggesting that *NtNRT1.1B* could be adopted as a potential molecular target aiming at the improvement of crop N-use efficiency.

## Data availability statement

The original contributions presented in the study are included in the article/**Supplementary Material**. Further inquiries can be directed to the corresponding authors.

## Author contributions

CW performed major experiments. YX, PH, MZ, MF, WY, WL, and FC participated in the experiments including cloning, tobacco growth, transgenic plant generation, and physiological analysis. L-HL, CW, WP, and SD designed the experiments and prepared/discussed the manuscript. All authors read and approved the final manuscript.
