# Supplementary figures and images for: Molecular identification and physiological functional analysis of NtNRT1.1B that mediated nitrate long-distance transport and improved plant growth when overexpressed in tobacco
Source: Front Plant Sci. 2023 Feb 28;14:1078978. doi: 10.3389/fpls.2023.1078978 (PMC10011135; doi:10.3389/fpls.2023.1078978)

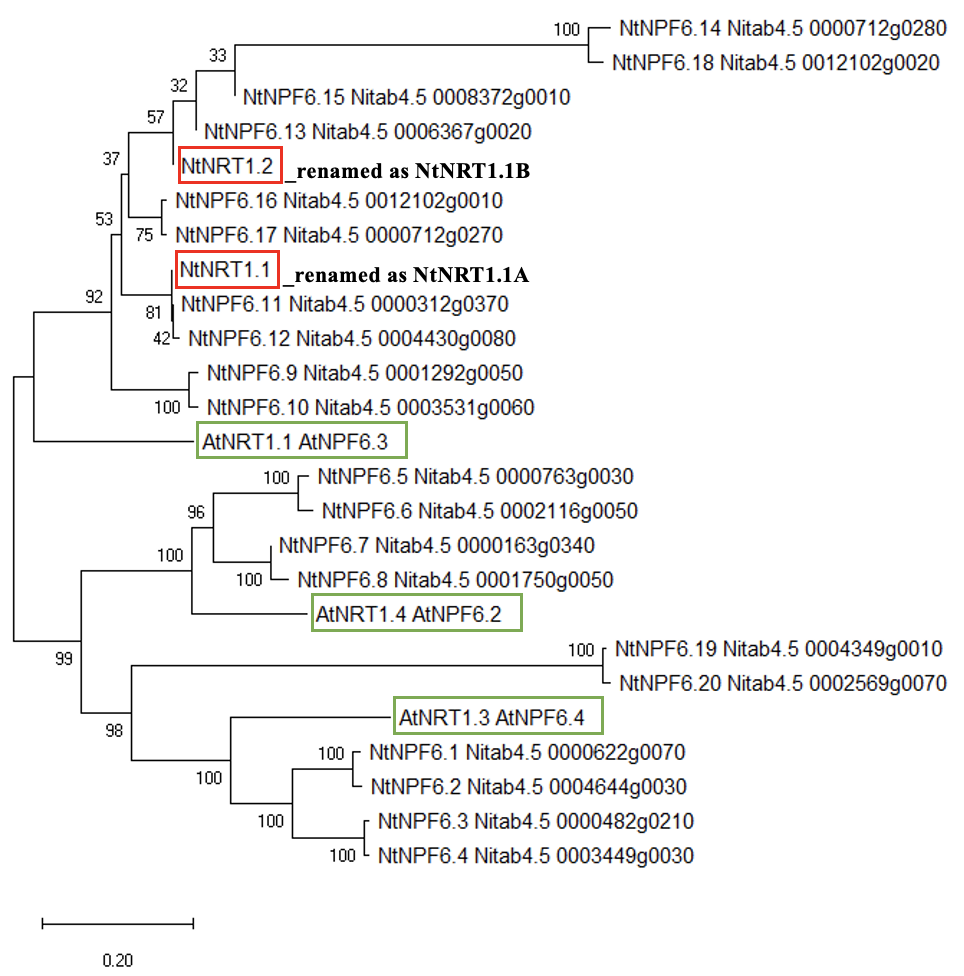

Supplement: Supplementary file 1 [file Image_1.png]

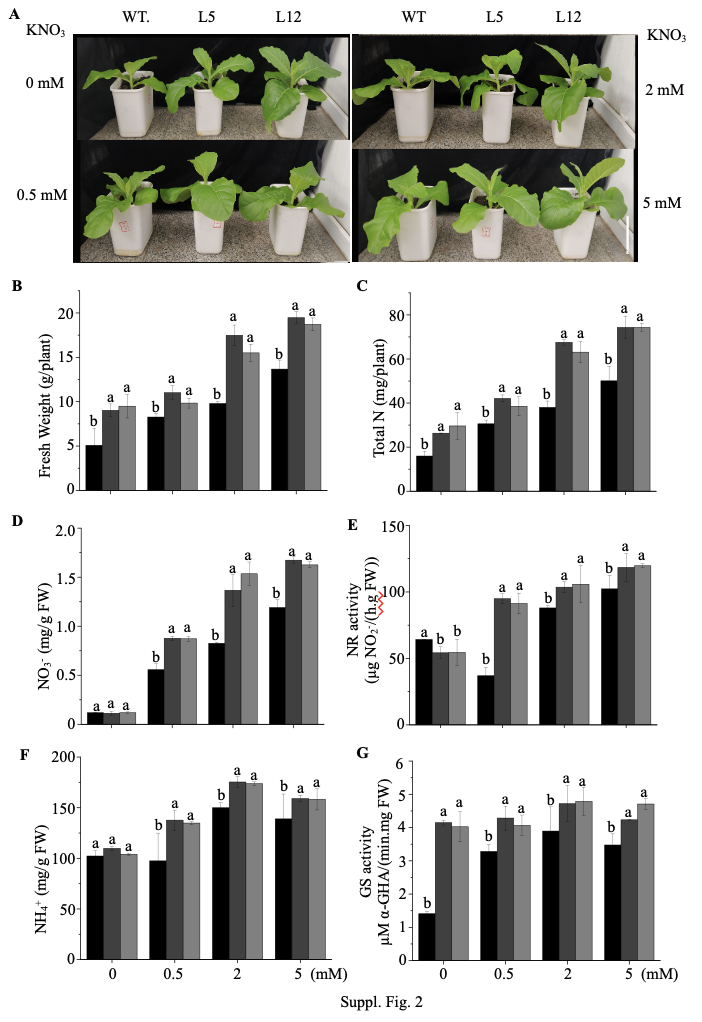

Supplement: Supplementary file 2 [file Image_2.png]

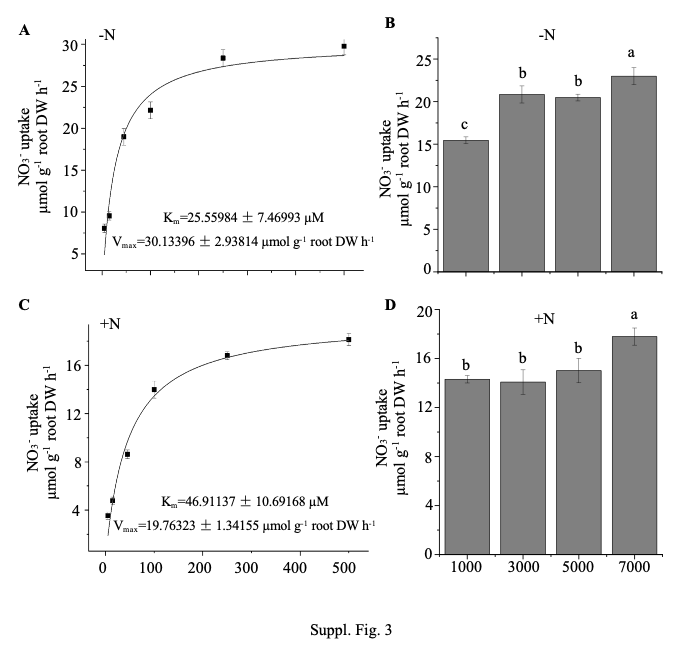

Supplement: Supplementary file 3 [file Image_3.png]
