# Supplementary material for: Molecular identification and physiological functional analysis of NtNRT1.1B that mediated nitrate long-distance transport and improved plant growth when overexpressed in tobacco
Source: Front Plant Sci. 2023 Feb 28;14:1078978. doi: 10.3389/fpls.2023.1078978 (PMC10011135; doi:10.3389/fpls.2023.1078978)
Supplement: Supplementary file 4 [file Table_1.docx]

**Supplemental Figure 1.** Phylogenetic tree of tobacco NtNRT1.1A and NtNRT1.1B with recent reported NtNPF6s and their representative counterparts from *Arabidopsis*. Genetic lineage analysis was conducted using the Neighbor-Joining method from MEGA7.0. Bootstrap values are from 1000 replications. Evolutionary distances were estimated in a unit of the number of amino-acid substitutions per site, with a scale bar equivalent to 0.2 substitutions per site. The numbers at each node are bootstrap values. Tobacco NtNPF6s gained from Zhang et al. (2022). The red and green line squared parts respectively indicate NtNRT1.1 and NtNRT1.2 cloned by Liu et al. (2018), *Arabidopsis* AtNPF6s extracted from “Aramemnon” (see Materials and Methods).

**Supplemental Figure 2.** *NtNRT1.2*-overexpressing improves the growth of tobacco in pot-soil conditions.

After seed germination (14 d) and pre-culture (10 d), similar size plants grown for 25 d in the pot-soil were supplied 3 times with 100 ml water containing NO− 3 at 4 different concentrations (i.e. 0, 0.5, 2 or 5 mM) (see Materials and Methods). The measurement of N-related physiological components followed the same protocol as did for Figure 6 (see Materials and Methods). Four biological replicates were conducted for each treatment. (**A**) Pictures of representative growth phenotype of *NtNRT1.2*-overexpressing lines (L5, L12) and WT tobacco in pot-soil. (**B-G**) Quantification of fresh weight of shoots and roots (B), total N (C), content of NO− 3 (D) and NH+ 4 (F), enzymatic activity of glutamine synthetase GS (E) and nitrate reductase NR (G). Means +SD (n =4) were depicted, and different letters indicate statistically significant differences (P < 0.05, one-way ANOVA).

**Supplemental Figure 3.** Measurement of concentration-dependent short-term NO_3_^-^-uptake by roots of *NtNRT1.1B*-overexpressing tobacco and its corresponding WT K326. The plant growth and root uptake assay experiment are the same as described for figure 6B. The *NtNRT1.1B*-transgenic line 5 was used.
